# Prognostic value of lncRNA ROR expression in various cancers: a meta-analysis

**DOI:** 10.1042/BSR20181095

**Published:** 2018-09-28

**Authors:** Renfu Lu, Junjian Chen, Lingwen Kong, Hao Zhu

**Affiliations:** Department of Cardiothoracic Surgery, Chongqing Emergency Medical Center, The Affiliated Central Hospital of Chongqing University, Chongqing 400014, China

**Keywords:** cancer, lncRNA ROR, meta-analysis, prognosis

## Abstract

**Background:** There is a dispute on the prognostic value of long non-coding RNA regulator of reprogramming (lncRNA ROR) in cancers. The purpose of the present study was to evaluate the prognostic significance of lncRNA ROR expression in human cancers. **Methods:** PubMed, Embase, and Cochrane Library were searched to look for relevant studies. The meta-analyses of prognostic and clinicopathological parameters (CPs) were conducted. **Results:** A total of ten studies were finally included into the meta-analysis. High lncRNA ROR expression was significantly associated with shorter overall survival (hazard ratio [HR] = 2.88, 95% confidence interval [CI] = 2.16–3.84, *P*<0.01) and disease-free survival (HR = 3.25, 95% CI = 2.30–4.60, *P*<0.01) compared with low lncRNA ROR expression. Besides, high lncRNA ROR expression was obviously related to more advanced clinical stage (*P*<0.01), earlier tumor metastasis (*P*=0.02), lymph node metastasis (*P*<0.01), and vascular invasion (*P*<0.01) compared with low lncRNA ROR expression. However, there was no significant correlation between lncRNA ROR expression and other CPs, including age (*P*=0.18), gender (*P*=0.33), tumor size (*P*=0.25), or tumor differentiation (*P*=0.13). **Conclusion:** High lncRNA ROR expression was associated with worse prognosis in cancers. LncRNA ROR expression could serve as an unfavorable prognostic factor in various cancers.

## Introduction

Despite great advancements in detection, surgical resection, chemotherapy, radiotherapy, and multidisciplinary treatments, cancer is still a critical health problem and major cause of mortality worldwide [1,2]. In view of the poor prognosis of cancer patients, a growing number of researchers begin to look for optimal prognostic biomarkers for cancers [3,4]. However, the sensitivity and specificity of current tumor biomarkers are not very desirable.

Long non-coding RNAs (lncRNAs) refer to non-protein coding RNAs that are greater than 200 nucleotides [5]. LncRNAs are involved with multiple diseases such as heart diseases, genetic diseases and cancers, especially cancers [6,7]. Increasing evidence has testified that lncRNAs play a critical role in the tumorigenesis, invasion, and metastasis of human cancers [8–10]. Many lncRNAs have been proved to associate with the prognosis of human cancers, such as metastasis-associated lung adenocarcinoma transcript 1 (MALAT1) [3], HOXA transcript at the distal tip (HOTTIP) [4], and growth arrest-specific transcript 5 (GAS5) [11].

LncRNA regulator of reprogramming (lncRNA ROR) is a type of lncRNAs, which has been identified as a promoter of human-induced pluripotent stem cells and participates miRNA-mediated suppression in human embryonic stem cell self-renewal [12]. Recently, several studies manifested that lncRNA ROR might affect the prognosis of cancers; however, the definite conclusion has not been researched on account of the controversial results among different studies. Gao et al. [13] declared that there was no obvious correlation between lncRNA ROR expression and clinical stage in pancreatic cancer. Similar results were observed in Wang et al. [14] study focusing on gallbladder cancer. Nevertheless, Qu et al. [15] and Shi et al. [16] found that high lncRNA ROR expression predicted more advanced clinical stage compared with low lncRNA ROR expression in lung and renal cancers, respectively. Gao et al. [13] failed to detect the distinct relationship between lncRNA ROR expression and lymph node metastasis in pancreatic cancer. However, Liu et al. [17] found that high lncRNA ROR expression was obviously associated with earlier lymph node metastasis in esophageal cancer. In view of these controversial results, this meta-analysis was performed to explore the prognostic value of lncRNA ROR expression in various cancers.

## Materials and methods

The present study was performed in compliance with Preferred Reporting Items for Systematic reviews and Meta-Analyses (PRISMA) [18].

### Search strategy

PubMed, Embase, and Cochrane Library were comprehensively searched up to July 24, 2018. The search strategy was as follows: (‘tumor’ OR ‘cancer’ OR ‘neoplasm’ OR ‘carcinoma’) AND (‘lincRNA ROR’ OR ‘lncRNA ROR’ OR ‘long non-coding RNA ROR’ OR ‘long intergenic non-coding RNA ROR’ OR ‘long intergenic non-coding RNA regulator of reprogramming’). There was no restriction on the language. We also checked the references of retrieved articles to avoid missing relative studies.

### Inclusion and exclusion criteria

The study would be included in this meta-analysis if it met the following criteria: (I) patients were diagnosed with cancers; (II) lncRNA ROR expression level was detected; (III) patients were divided into two groups based on the lncRNA ROR expression level; (IV) efficient data were provided; (V) full-text was available. The following studies would be excluded from this meta-analysis: duplicated publications or patients, reviews, case reports, letters, comments, animal experiments, cell experiments, or studies without efficient data.

### Data extraction and quality assessment

Two authors extracted the data and assessed the quality of included studies independently. Any disagreement during this process was resolved by group discussion. The following variables were extracted: the first author, publication year, number of patients, cut-off value of lncRNA ROR expression level, analysis model, and clinical outcomes. The hazard ratio (HR) and corresponding 95% confidence interval (CI) of overall survival (OS) or disease-free survival (DFS) were directly or indirectly extracted from included studies according to Tierney et al. [19] study. The quality of studies was evaluated using Newcastle–Ottawa Scale (NOS). Studies were considered to be of high quality when NOS was equal to or greater than six [20].

### Statistical analysis

All analysis was conducted with Review Manager 5.3 (The Cochrane Collaboration, Copenhagen, Denmark) and Stata 12.0 (Stata, College Station, TX) for Windows. For OS and DFS, HR and corresponding 95% CI were used as the summary measures. While for clinicopathological parameters (CPs), odds ratio (OR) and corresponding 95% CI were applied. Besides, inter-study heterogeneity was assessed using Chi-squared test and *I*^2^ statistic. The *I*^2^ ≤ 50% or *P* value for heterogeneity >0.10 showed that there was no obvious heterogeneity among studies, as a result, a fixed-effect model should be utilized. If not, a random-effect model should be applied. Funnel plots, Begg’s test, and Egger’s test were performed to evaluate the publication bias. Sensitivity analysis was also conducted to check the stability of results. The association was considered to be significant when *P*<0.05.

## Results

### Literature search and selection

**Figure 1:**
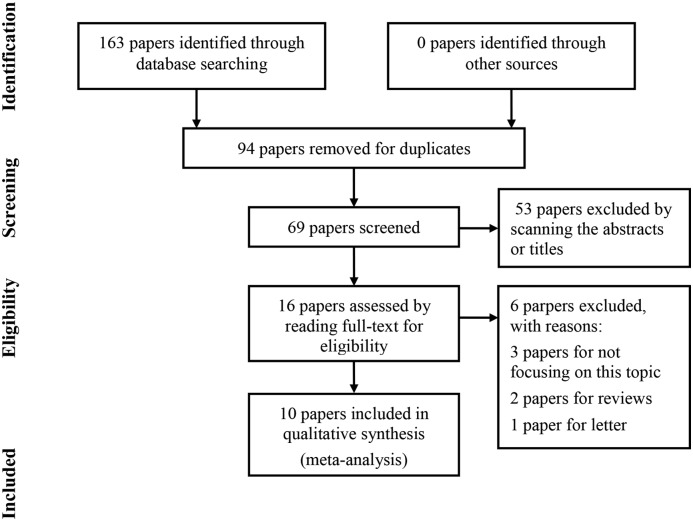
The flow chart of literature search and selection

As shown in Figure 1, a total of 163 papers were initially retrieved. Ninety-four papers were removed for duplicates. Then, 53 papers were directly excluded by scanning titles or abstracts. The full-text of the remaining 16 papers were carefully read and 6 papers were excluded for the following reasons: three papers were irrelevant to the interested topic, two papers were reviews, and one paper was a letter. Ten studies were finally included into this meta-analysis [13–17,21–25].

### Characteristics of included studies

The characteristics of included studies were listed in Table 1. A total of ten studies were ultimately included into the present study [13–17,21–25]. The sample size varied from 30 to 229 among included studies. The percentage of male varied from 30.00 to 86.11% in eight studies [13–17,21,23,25]. The expression level of lncRNA ROR was detected by quantitative real-time polymerase chain reaction (qRT-PCR) in all studies. Additionally, the percentage of patients with high lncRNA ROR expression level varied from 37.20 to 53.33% among all studies. With respect to clinical outcomes, all studies reported OS [13–17,21–25], nine studies reported CPs [13–17,21–23,25] and four studies reported DFS [14,15,17,23]. Besides, eight kinds of cancers were analyzed in this study, including pancreatic cancer [13,22], non-small-cell lung cancer (NSCLC) [15,24], gallbladder cancer [14], colon cancer [25], bladder cancer [21], hepatocellular carcinoma (HCC) [23], esophageal squamous cell carcinoma (ESCC) [17], and renal cancer [16]. Regarding the analysis model, the correlation between lncRNA ROR expression and OS was assessed with multivariate analysis model in four studies [15,17,21,25] and univariate analysis model in six studies [13,14,16,22–24]. The adjusted factors in the multivariate analysis of OS were listed in Supplementary Table S1. The NOS score was equal to or greater than six in all the studies.

**Table 1:** The characteristics of included studies

Study	Sample size (*n*)	Male (*n*, %)	Detection method	Cut-off value	High expression (*n*, %)	Outcome	Cancer type	Analysis	NOS
Wang (2016) [14]	30	9 (30.00%)	qRT-PCR	NA	14 (46.66%)	CP, OS, DFS	Gallbladder cancer	U	6
Zhou (2016) [25]	60	33 (55.00%)	qRT-PCR	Median value	32 (53.33%)	CP, OS	Colon cancer	M	7
Gao (2016) [13]	51	32 (62.70%)	qRT-PCR	NA	19 (37.20%)	CP, OS	Pancreatic cancer	U	6
Chen (2017) [21]	36	31 (86.11%)	qRT-PCR	CTNAT	18 (50.00%)	CP, OS	Bladder cancer	M	8
Fu (2017) [22]	81	NA	qRT-PCR	NA	41 (50.61%)	CP, OS	Pancreatic cancer	U	6
Li (2017) [23]	88	67 (76.14%)	qRT-PCR	CTNAT	44 (50.00%)	CP, OS, DFS	HCC	U	6
Liu (2017) [17]	120	56 (46.67%)	qRT-PCR	NA	64 (53.33%)	CP, OS, DFS	ESCC	M	8
Qu (2017) [15]	229	112 (48.90%)	qRT-PCR	Median value	113 (49.34%)	CP, OS, DFS	NSCLC	M	7
Shi (2017) [16]	36	21 (58.33%)	qRT-PCR	CTNAT	19 (52.78%)	CP, OS	Renal cancer	U	6
Xia (2017) [24]	40	NA	qRT-PCR	Median value	NA	OS	NSCLC	U	6

Abbreviations: CTNAT, compared to non-tumor adjacent tissues; M, multivariate; NA, not available; U, univariate.

### Meta-analysis of OS

**Figure 2:**
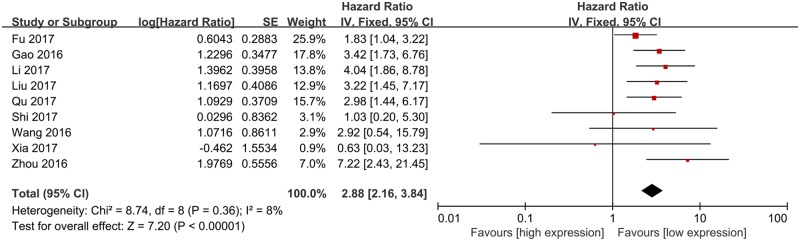
The meta-analysis of OS

All studies assessed the correlation between lncRNA ROR expression and OS in human cancers. However, Chen et al. [21] failed to provide the sufficient data to obtain the HR and corresponding 95% CI of OS; therefore, nine studies were ultimately included into the meta-analysis of OS. As shown in Figure 2, no significant heterogeneity was observed among studies and a fixed-effect model was used (*I*^2^ = 8%). The results indicated that high lncRNA ROR expression was obviously related to shorter OS compared with low lncRNA ROR expression in various cancers (HR = 2.88, 95% CI = 2.16–3.84, *P*<0.01). To further explore the association between lncRNA ROR expression and OS, subgroup analyses based on analysis model, sample size, and cancer type were conducted. As listed in Table 2, high lncRNA ROR expression was obviously correlated with shorter OS compared with low lncRNA ROR expression in all subgroup analyses (*P*<0.01).

**Table 2:** The subgroup analysis for the association between lncRNA ROR expression and OS

Variables	Included studies	HR 95% CI	*P*	*I*^2^	Model
**Analysis model**					
Multivariate	3	3.65 [2.25–5.91]	<0.01^*^	0%	Fixed
Univariate	6	2.52 [1.76–3.61]	<0.01*	7%	Fixed
**Sample size**					
<60	4	2.72 [1.52–4.85]	<0.01*	0%	Fixed
≥60	5	2.93 [2.10–4.08]	<0.01*	34%	Fixed
**Cancer type**					
Digestive cancers	6	3.02 [2.19–4.17]	<0.01*	19%	Fixed
NSCLC	2	2.74 [1.35–5.56]	<0.01*	0%	Fixed

*, The association between lncRNA ROR expression and OS was considered to be significant when *P*<0.05.

### Meta-analysis of DFS

**Figure 3:**
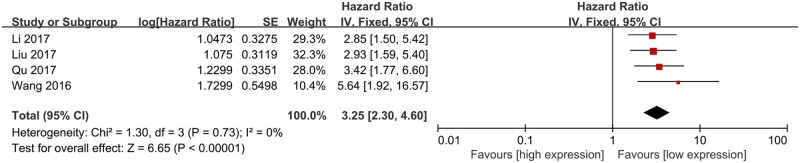
The meta-analysis of DFS

As shown in Figure 3, significant association between lncRNA ROR expression and DFS was detected, and patients with high lncRNA ROR expression tended to have shorter DFS compared with those with low lncRNA ROR expression (HR = 3.25, 95% CI = 2.30–4.60, *P*<0.01). There was no heterogeneity among studies and a fixed-effect model was applied (*I*^2^=0%).

### Meta-analysis of CPs

The associations between lncRNA ROR expression and CPs were analyzed and listed in Table 3. The results demonstrated that high lncRNA ROR expression was significantly related to more advanced clinical stage (*P*<0.01), earlier tumor metastasis (*P*=0.02), lymph node metastasis (*P*<0.01), and vascular invasion (*P*<0.01). However, no obvious relationship between lncRNA ROR expression and other CPs was observed, including age (*P*=0.18), gender (*P*=0.33), tumor size (*P*=0.25), or tumor differentiation (*P*=0.13).

**Table 3:** The meta-analysis for the association between lncRNA ROR expression and CPs

Variables	Included studies	Patients (*n*)	OR 95% CI	*P*	*I*^2^	Model
Age (old compared with young)	9	721	1.22 [0.91–1.65]	0.18	0%	Fixed
Gender (male compared with female)	8	650	1.18 [0.85–1.62]	0.33	0%	Fixed
Clinical stage (III/IV compared with I/II)	6	536	3.45 [1.64–7.14]	<0.01*	67%	Random
Tumor size (large compared with small)	6	564	1.50 [0.75–3.00]	0.25	69%	Random
Tumor metastasis (yes compared with no)	3	385	4.45 [1.33–14.89]	0.02*	76%	Random
Tumor differentiation (poor compared with well)	3	241	0.66 [0.39–1.12]	0.13	0%	Fixed
Lymph node metastasis (yes compared with no)	5	534	3.10 [2.10–4.57]	<0.01*	55%	Random
Vascular invasion (yes compared with no)	2	148	3.40 [1.73–6.68]	<0.01*	0%	Fixed

‡, The association between lncRNA ROR expression and CPs was considered to be significant when *P*<0.05.Abbreviation: NA, not available.

### Publication bias and sensitivity analysis

**Figure 4:**
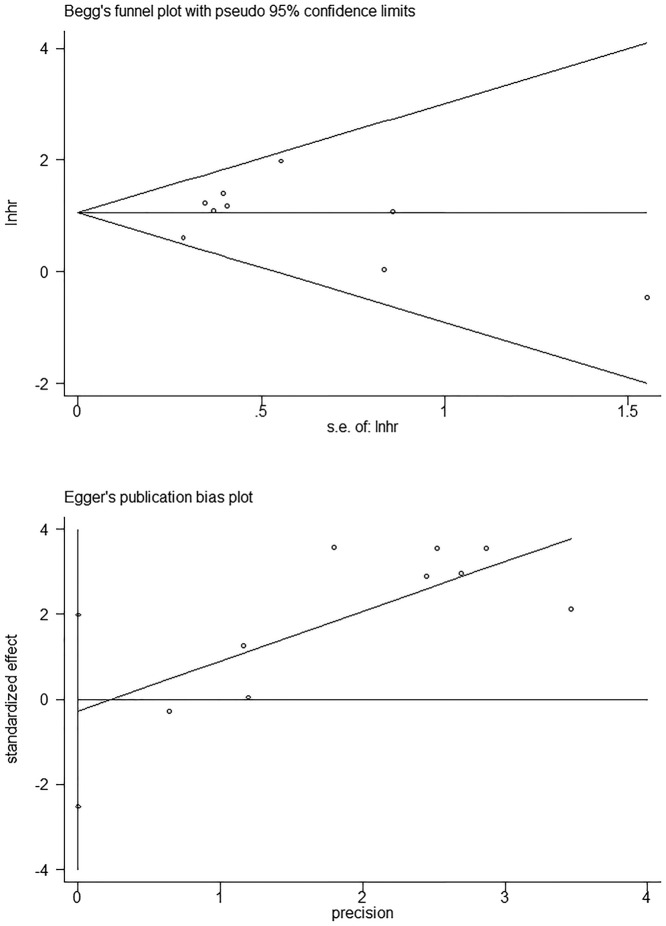
The detection of publication bias for meta-analysis of OS

**Figure 5:**
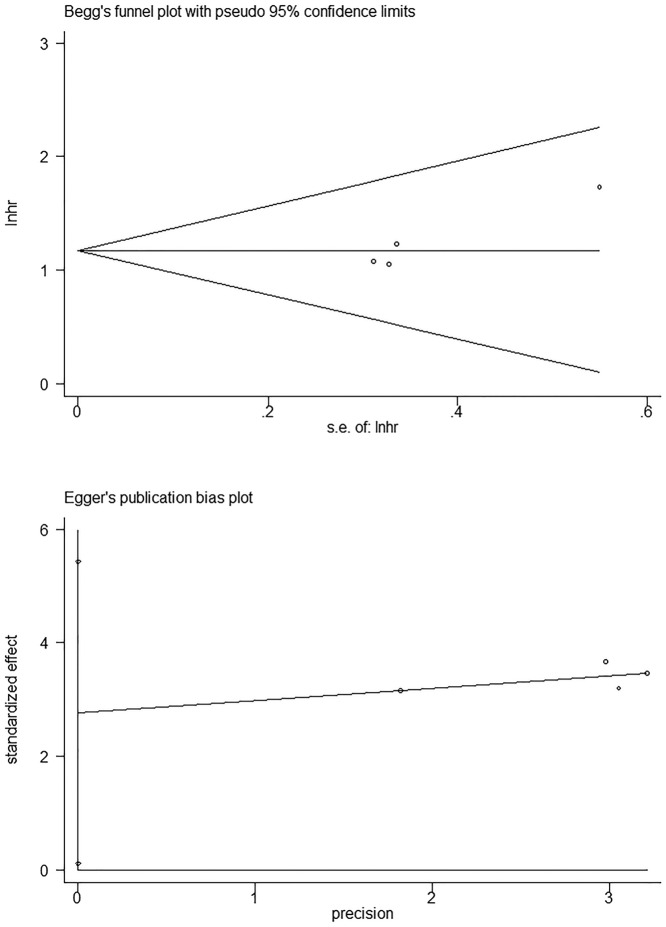
The detection of publication bias for meta-analysis of DFS

**Figure 6:**
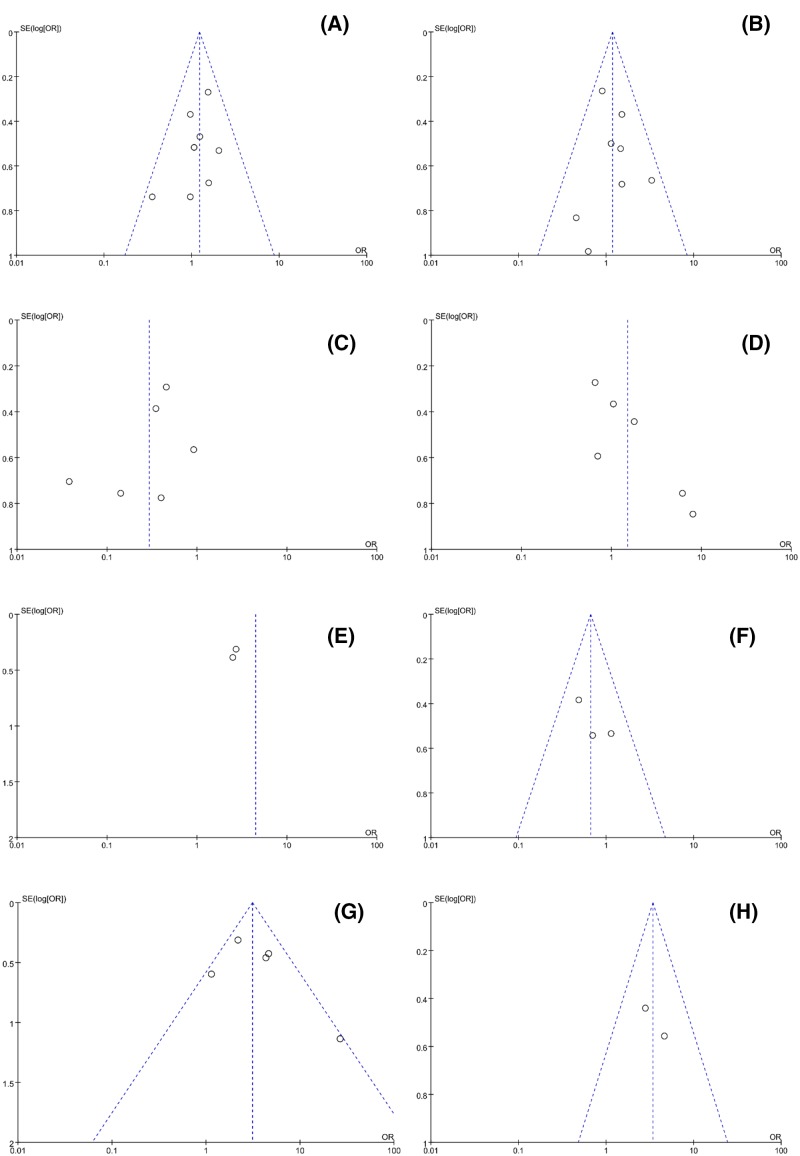
The detection of publication bias for meta-analyses of CPs (**A**) age; (**B**) gender; (**C**) clinical stage; (**D**) tumor size; (**E**) tumor metastasis; (**F**) tumor differentiation; (**G**) lymph node metastasis; (**H**) vascular invasion.

**Figure 7:**
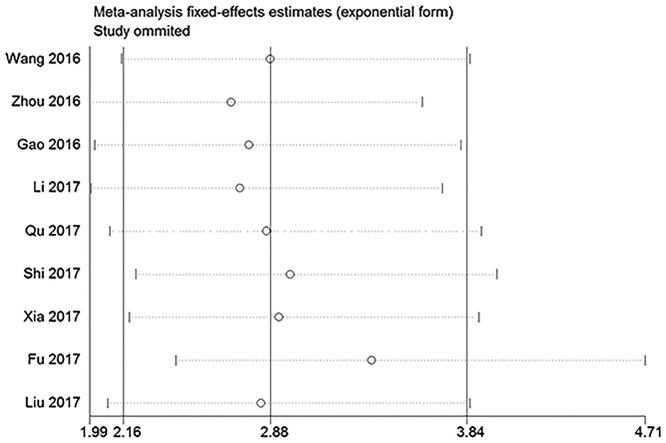
The sensitivity analysis for meta-analysis of OS

**Figure 8:**
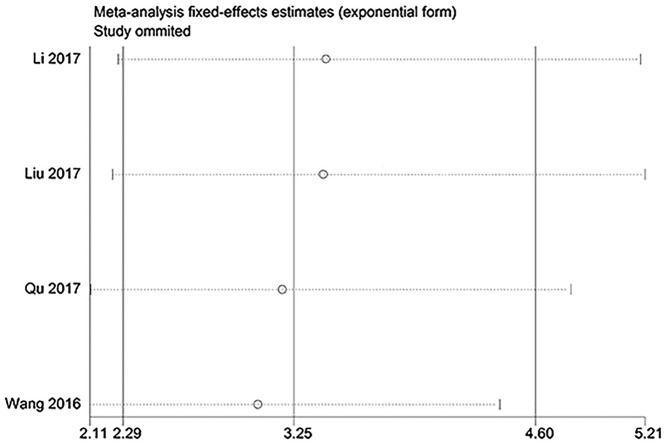
The sensitivity analysis for meta-analysis of DFS

With respect to OS, there was no significant publication bias based on Begg’s test (*P*=0.92) and Egger’s test (*P*=0.79) in (Figure 4). Similarly, regarding to DFS, no obvious publication bias was observed according to Begg’s test (*P*=0.31) and Egger’s test (*P*=0.05) (Figure 5). Besides, there was no obvious publication bias in terms of CPs (Figure 6). Sensitivity analysis for OS (Figure 7) and DFS (Figure 8) was conducted to test the robustness of results.

## Discussion

LncRNAs have been proved to play a critical role in tumorigenesis, invasion, and metastasis of cancers [9,10]. Among lncRNAs, the human lncRNA ROR, 2.6 kb in length and previously identified as a ‘regulator of reprogramming’, was involved with the process of reprogramming differentiated cells into induced pluripotent stem cells [26,27]. Recently, lncRNA ROR expression was supposed to be related to prognosis in several cancers, including lung cancer [24], pancreatic cancer [22], and bladder cancer [21], but controversial results were found. Here, for the first time, we conducted this meta-analysis to summarize the prognostic value of lncRNA ROR expression in human cancers.

In the present study, we discovered that high lncRNA ROR expression was significantly associated with shorter OS and DFS compared with low lncRNA ROR expression, and the subgroup analyses of OS observed similar results. As for CPs, high lncRNA ROR expression was significantly related to more advanced clinical stage, earlier tumor metastasis, lymph node metastasis, and vascular invasion compared with low lncRNA ROR expression. Therefore, our study demonstrated that high lncRNA ROR expression might be an unfavorable prognostic factor in various cancers. Similarly, Zhang et al. [28] study also discovered that high lncRNA ROR expression might contribute to the lymph node metastasis in breast cancer (*P*=0.046). It should be noted that we failed to observe the statistical association between lncRNA ROR expression and tumor size, which might be explained that tumor size was not always related to cancer prognosis. Besides, small sample size might also contribute to this negative finding. Furthermore, our study also found that there was no evident association between lncRNA ROR expression and tumor differentiation. Nevertheless, only three studies were included into the analysis of tumor differentiation, which might lower the reliability of results. Differently, Arunkumar et al. [29] found that lncRNA ROR expression was significantly associated with cellular differentiation in oral cancer (*P*<0.05), which was not analyzed in our study for insufficient data. Therefore, more studies should be carried out to explore the association between lncRNA ROR expression and clinicopathological variables in cancers.

Although many studies have testified the prognostic value of lncRNA ROR in cancers, the underlying mechanism remains indistinct. In general, lncRNA ROR participated in diverse biological processes including proliferation, differentiation, invasion, and metastasis of human cancers. Eades et al. [30] tried exploring the prognostic role of lncRNA ROR in triple-negative breast cancer, and they discovered that lncRNA ROR and miR-145 might regulate the tumor invasion via targeting the ARF6. Zhang et al. [31] found that lncRNA ROR regulated the expression of miR-205, ZEB1 and ZEB2, and further inhibited the EMT of breast cancer cells and enhanced the sensibility of breast cancer cells to tamoxifen. Besides, Li et al. [32] verified that the inhibition of lncRNA ROR could reverse resistance to tamoxifen by inducing autophagy in breast cancer. As for pancreatic cancer, lncRNA ROR regulated Nanog expression by sponging miR-145 and further induced poor prognosis [13]. Similarly, Liu et al. [33] also discovered that miR-145 and lncRNA ROR were involved with the invasion of pancreatic cancer. Li et al. [34] study showed lncRNA ROR conferred gemcitabine resistance to pancreatic cancer cells at least partly via inducing autophagy, they further came up with lncRNA ROR/miR-124/PTBP1/PKM2 axis, which might play an important role in the regulation of gemcitabine resistance in pancreatic cancer cells. Moreover, Yang et al. [35] demonstrated that lncRNA ROR promoted the resistance of radiotherapy by targeting the p53/miR-145 pathway in colorectal cancer cells. Although lncRNA ROR acted as an oncogene in several cancers, Feng et al. [36] found that lncRNA ROR could inhibit the proliferation of cancer cells and self-renewal of glioma stem cells partly by inhibiting the KLF4 expression. In view of limited investigations, more researches should be carried out to explore the underlying mechanism of prognostic value of lncRNA ROR in cancers.

There were several highlights of our study. First, to our knowledge, the present study was the first meta-analysis to explore the prognostic value of lncRNA ROR expression in cancers. Second, comprehensive analyses of prognostic and CPs were conducted in the present study, which further confirmed the unfavorable prognostic role of high lncRNA ROR expression in cancers. Third, our study strictly followed the rules of PRISMA [18]; therefore, the methodology was normative. Fourth, the heterogeneity in the analysis of OS and DFS was very slight, which guaranteed the accuracy of results. Nonetheless, our study was not without limitations. First, there were only ten studies in this meta-analysis, the relatively small sample size might reduce the reliability of the results. Second, the cut-off value varied a lot among included studies, which might limit the clinical application of the conclusion. Third, although there was no restriction on countries during the process of literature selection, all included studies were conducted in China. As a result, the conclusion might be hard to be extended to other countries. Therefore, more studies with high quality and a large population should be carried out to clarify this issue.

## Conclusion

High lncRNA ROR expression was associated with shorter OS and DFS in various cancers. Besides, high lncRNA ROR expression was related to more advanced clinical stage, earlier tumor metastasis, lymph node metastasis, and vascular invasion compared with low lncRNA ROR expression. Therefore, lncRNA ROR expression could serve as a prognostic factor in various cancers.

## Supporting information

**Supplementary Table 1:** The adjusted factors in the multivariate analysis of OS.

## Abbreviations

CI: confidence interval
CP: clinicopathological parameter
DFS: disease-free survival
ESCC: esophageal squamous cell carcinoma
HCC: hepatocellular carcinoma
HR: hazard ratio
lncRNA: long non-coding RNA
lncRNA ROR: long non-coding RNA regulator of reprogramming
NOS: Newcastle–Ottawa Scale
NSCLC: non-small-cell lung cancer
OS: overall survival
OR: odds ratio
PRISMA: Preferred Reporting Items for Systematic reviews and Meta-Analyses
qRT-PCR: quantitative real-time polymerase chain reaction
