# Supplementary material for: Prognostic value of lncRNA ROR expression in various cancers: a meta-analysis
Source: Biosci Rep. 2018 Sep 28;38(5):BSR20181095. doi: 10.1042/BSR20181095 (PMC6165833; doi:10.1042/BSR20181095)
Supplement: Supplementary file 1 [file bsr20181095_Supp1.pdf]

## Supplementary Table 1 The adjusted factors in the multivariate analysis of OS.

| | LincRNA -ROR | Age | Gender | Tumor location | T stage | Clinical stage | Lymph nodes metastasis | Metastasis | Tumor size | Tumor differentiation | Smoking |
|---|---|---|---|---|---|---|---|---|---|---|---|
| Zhou 2016 [25] | √ | √ | √ | √ | √ | √ | √ | | | √ | |
| Chen 2017 [21] | √ | √ | √ | | √ | √ | √ | √ | | | √ |
| Liu 2017 [17] | √ | √ | √ | √ | | √ | √ | √ | √ | √ | |
| Qu 2017[15] | √ | √ | √ | | | √ | √ | √ | √ | | |

OS, overall survival.
